# Reward pursuit during a translational reward task correlates with anhedonia reductions following rTMS in patients with major depressive disorder

**DOI:** 10.1038/s41398-026-04248-3

**Published:** 2026-07-10

**Authors:** Samantha V. Abram, Aaron N. McInnes, Christi R. P. Sullivan, Dawson C. Cooper, Brian M. Sweis, Alik S. Widge

**Affiliations:** 1 Mental Health Service, San Francisco Veterans Affairs Healthcare System, San Francisco, CA USA; 2 Department of Psychiatry & Behavioral Sciences, University of California, San Francisco, CA USA; 3 Department of Psychiatry & Behavioral Sciences, University of Minnesota Twin Cities, Minneapolis, MN USA; 4 Department of Psychiatry & Neuroscience, Mount Sinai, New York, NY USA

**Keywords:** Human behaviour, Depression

## Abstract

People with major depressive disorder (MDD) often struggle to pursue previously rewarding activities, which may relate to deficits experiencing pleasure, termed anhedonia. Anhedonia remains a challenge to target and treat. Repetitive transcranial magnetic stimulation (rTMS) alleviates anhedonia in some patients with MDD, though rTMS is logistically burdensome. Identifying novel, translational phenotypes of reward pursuit may help identify who is most likely to respond to treatment. We used a translational reward task (Web-Surf) to test whether reward pursuit strategies in patients with MDD correlate with anhedonia reductions following rTMS. A naturalistic sample of patients with treatment-resistant MDD (*N* = 32) underwent daily rTMS and completed Web-Surf weekly. Depression and anhedonia symptoms were measured with the Patient Health Questionnaire (PHQ-9). We examined whether the number of rewards earned, at baseline, correlated with self-reported hedonic experience and anhedonia reductions following rTMS. We also tested whether changes in reward earnings, over the rTMS course, were specific to patients with reduced anhedonia. Patients with MDD who earned more desired rewards at baseline reported a more pleasurable experience during Web-Surf (baseline) and exhibited greater anhedonia reductions (late treatment – baseline). Patients increasingly earned more desired rewards over the therapy course, and this change was specific to patients whose anhedonia improved with rTMS. We present a novel translational reward-pursuit metric that correlates with anhedonia reductions following rTMS therapy. This translational phenotype shows promise for tracking clinical treatment response in depression.

## Introduction

Every day people make thousands of decisions about whether to engage with known rewards or search for unknown alternatives. People with major depressive disorder (MDD) often struggle to pursue previously rewarding activities, which may relate to underlying impairments in the ability to experience pleasure, termed *anhedonia* [1–3]. Anhedonia is a debilitating symptom with a complex pathophysiology [4]. While originally viewed as a deficit in consummatory pleasure, there is growing evidence that other reward processes also contribute to anhedonia [3, 5]. Repetitive transcranial magnetic stimulation (rTMS) can yield large reductions in depression symptoms, including anhedonia [6, 7]. However, rTMS response is variable [8], which underscores the need for biomarkers of treatment response [9]. Identifying clear, translational phenotypes relevant to anhedonia [4, 5] could help clarify who will benefit most from rTMS and inform target selection and/or treatment titration. Accept/reject reward-related decisions are ripe for cross-species translation [10] and capture how well humans or rodents continue to pursue previously enjoyable rewards. Here we examine whether accept/reject reward-related decisions correlate with anhedonia reductions in patients with MDD undergoing rTMS therapy.

Accept/reject decisions can be modeled through the lens of foraging theory which describes how animals optimize behavior when seeking nourishment [11]. These evolutionary-rooted decisions are similarly captured from parallel, translational foraging tasks called *Restaurant Row* and *Web-Surf* [10]. Restaurant Row is an animal foraging task with serial accept/reject decisions [12]. Animals have a fixed amount of time to cycle between four feeders, each providing a different flavored pellet (plain, chocolate, banana, cherry) after a variable, short delay; essentially, animals forage for their primary food source while on a limited time budget. Flavor preferences are stable across days and differ between animals [12, 13]. Web-Surf is a human foraging task directly translated from Restaurant Row [14]. Web-Surf includes real-time delays and primary rewards woven into a computerized framework. Humans make accept/reject decisions to forage for rewarding video clips of different “flavors” (e.g., kittens, dance) available after short delays. Subjective value is measured by how long a human or rodent will wait for a particular flavor; agents typically wait longer for more preferred rewards and are more likely to reject offers for less preferred flavors. Flavor preference can be expressed based on how many videos/pellets are earned from that flavor. Web-Surf differs from other translational reward pursuit paradigms that measure subjective-value based on willingness to exert effort/work for abstract rewards [2]. An asset of Web-Surf is its inclusion of primary rewards: like animal models, real-time hedonic experiences can be directly linked with economic decisions. Accept/reject decisions are associated with self-reported reward enjoyment in humans without psychiatric disorders [14–17]. Recent work demonstrates the reliability of decision behaviors and hedonic responses during Web-Surf across repeated sessions [18].

Across repeated testing sessions of Restaurant Row, mice wait increasingly longer for (and earn) more preferred rewards and become more efficient at rejecting less preferred offers [19–21]. Rejection becomes a valuable tool for mice to maximize time spent pursuing more preferred options. There can be an opportunity cost to accepting less preferred outcomes, and rejection enables better discernment between reward types. The extent to which an agent learns to distinguish between reward options could likewise measure changes in reward optimization among humans during Web-Surf. People with MDD often lack the drive to pursue previously pleasurable rewards and the typical bias to approach more frequently rewarded stimuli [2, 22]. One hypothesis is that an insensitivity to available, known rewards could yield a heightened exploration of alternatives with unknown reward values [23, 24]. We can test this hypothesis in Web-Surf based on the frequency with which rewards are earned from more preferred categories, and how well earnings (based on preference) align with in-the-moment hedonic experiences (i.e., ratings after viewing primary, video rewards).

Accept/reject decisions recruit prefrontally-mediated cognitive systems [17, 25–27] that can be impaired in MDD [28]. The prefrontal cortex is central to accept/reject decision-making; it helps to override reflexive or habitual actions in the service of more flexible behaviors that align with thoughtful intentions [29–31]. Prefrontal cortex deficits are reliably documented in MDD [28, 32], and contribute to difficulties engaging the cognitive control systems needed for reward pursuit [3]. Anhedonia is also associated with prefrontal deficits [33]. rTMS is an FDA-approved depression treatment that targets the prefrontal cortex. rTMS emits magnetic pulses on the scalp to modulate cortical excitability and brain function [34]. This method shows promise for enhancing effortful reward pursuit in patients with MDD [35]. While rTMS is efficacious for treatment-resistant MDD [36, 37], it is limited by its slow response time and logistical burdens, particularly the need for daily sessions for over a month [38]. Variability in treatment outcomes may partly stem from heterogeneity within the MDD diagnosis [39]. Identifying biomarkers that predict rTMS treatment response for specific symptoms, like anhedonia, can help address these challenges.

We used a translational reward task, Web-Surf, to evaluate accept/reject reward pursuit as a biomarker of clinical response in patients with MDD undergoing daily rTMS therapy. We first assess whether basic decision behaviors and hedonic responses are present in patients with MDD during Web-Surf, as observed in non-psychiatric human samples [14–17]. We measure successful reward pursuit based on relative earnings of more (versus less) preferred videos. We examine whether reward pursuit patterns in patients with MDD, at baseline, correlate with a) hedonic enjoyment, and b) anhedonia symptom reductions (following rTMS). Lastly, we assess whether patients increasingly optimize their reward pursuit strategies over the treatment course like mice [19, 20], i.e., learn to earn more preferred and fewer less preferred rewards, particularly patients who report anhedonia reductions.

## Methods

### Sample characteristics

32 patients with treatment-resistant MDD completed this observational study (mean age = 50.47±17.29; 18 women/14 men; Table S1 contains psychiatric comorbidities; Table S2 contains medication data). Because of the naturalistic nature of this study, this was a convenience sample, with sample size based on the number of participants who could be recruited during the study period. Participants were recruited from an academic interventional psychiatry clinic in suburban Minnesota and completed study procedures in parallel with their standard clinical care. This study did not include randomization. rTMS eligibility was based on FDA-approved indications, defined by self-reported persistent depressive symptoms despite adequate dose-duration trials of at least four FDA-approved antidepressant medications (confirmed by medical record review). Further details on study recruitment and eligibility are found in Supplemental Materials.

### Ethics approval and consent to participant

All procedures contributing to this work comply with the ethical standards of the Declaration of Helsinki. All procedures were approved by the Institutional Review Board at the University of Minnesota (STUDY00010052). Written informed consent was obtained from all participants. Participant data is fully anonymized and not to be identified from the manuscript.

### rTMS procedures

All patients received rTMS of the dorsolateral prefrontal cortex five days per week (36 treatments in a full series), with a Figure-8 (*n* = 21) or H1 (*n* = 11) coil. Supplemental Materials contains details on stimulation site and stimulation frequency in Hz (Tables S3, S4). No major adverse events were reported. Some patients indicated mild intolerance of stimulation. Table S5 details the number of patients who underwent rTMS each week; these *ns* correspond to the number of available Web-Surf sessions at each timepoint. The Patient Health Questionnaire (PHQ-9) was collected as part of standard clinical care to quantify depression symptom severity; the instrument was completed 5 times per week and scores were averaged within each week [40]. The PHQ-9 is the standard instrument for rTMS therapy and is sensitive to clinical change [41]. For the purpose of this study, we interpret PHQ-9 scores as relative indices of depression symptom fluctuations rather than the 2-week severity estimates. Symptoms for most patients improved with rTMS (Fig S1; Supplemental Materials).

One patient was missing all baseline sessions (i.e., task runs before starting rTMS), two completed only baseline, and one completed baseline and only the first active rTMS session. Primary analyses were restricted to the first 7 weeks of rTMS, however, several patients continued rTMS beyond 7 weeks.

### Web-surf task

**Fig. 1:**
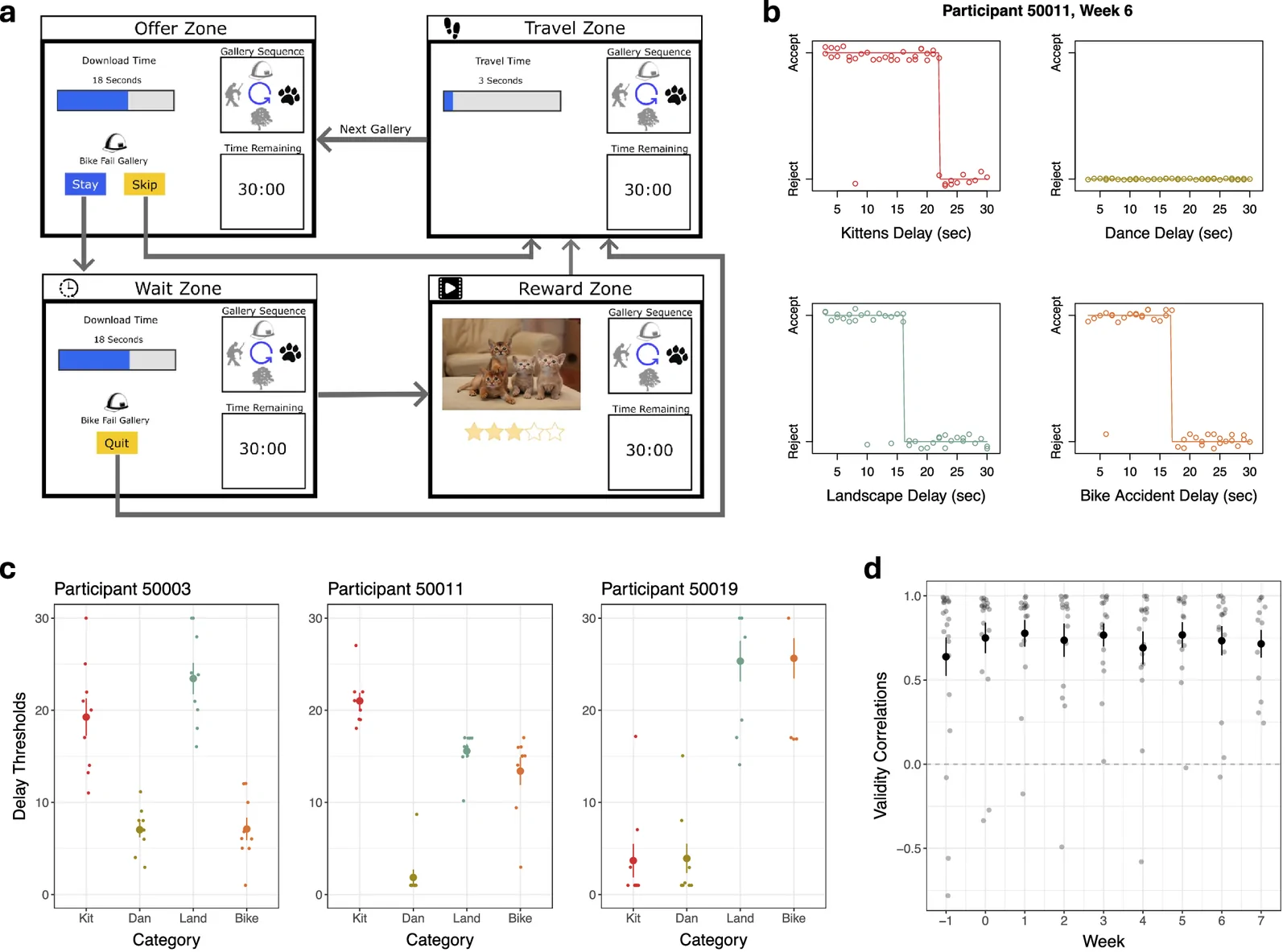
Web-Surf task captures reliable and valid decision behaviors in patients with major depressive disorder. **a** Layout of Web-Surf. Each trial begins in the Offer Zone where participants are presented with the time delay (3 – 30 secs) they must wait to obtain a video reward for the respective category. If they choose to wait (*accept*), they transition to the Wait Zone, where the delay begins to countdown. After the delay completes, a participant transitions to the Reward Zone where the video is presented, and they subsequently rate the video. Participants can also choose to *reject* offers in the Offer Zone or *quit* once the delay is counting down (in the Wait Zone). Regardless of decision (accept, reject, quit), they move ahead to the Travel Zone before arriving in the next Offer Zone. **b** Patients with major depressive disorder make economic choices based on the subjective value of video rewards. Example of a single session from one patient shows clear thresholds that differ by video category. **c** Preferences were consistent within each patient across time. Individual points represent data from a given week. **d** Category-specific delay thresholds were highly correlated with respective video ratings across testing sessions. Individual points represent correlation data from a given week. Data shown as the mean ± standard error of the mean across participants. Abbreviations: Kit kitten, Dan dance, Land landscape, Bike bike accidents.

Patients completed a weekly experimental testing battery that included Web-Surf. Experimental tasks were completed on a computer in the clinic space (see Supplemental Materials for more details). During Web-Surf, patients with MDD had a fixed amount of time ( ~ 30 min) to cycle between four video categories, each providing a different “flavor” of video rewards (kittens, dance, landscapes, bike accidents) after a variable delay (3–30 sec). The video reward available for each trial was a unique 4-sec clip; video rewards were thematically related within each category. The relative value of each video reward/category was solely based on subjective preferences (i.e., decisions). As illustrated in Fig. 1a, participants could: 1) *accept* an offer (in the Offer Zone) and wait through the delay and then watch the video (in the Reward Zone), 2) *reject* an offer (in the Offer Zone) and advance to the next category to receive a new offer, or 3) accept an offer and then *quit* (in the Wait Zone) after the delay started counting down. Additional task details are provided in Supplemental Materials. Due to clinic scheduling constraints, some Web-Surf sessions had shorter durations ( ~ 10–20 min). To determine whether this presented a statistical confound in our primary analyses, we confirmed that our results remained significant when restricting analyses to sessions at least 20 min long.

### Key study variables

#### Category-specific delay thresholds

Thresholds were calculated with a Heaviside step function where *accept* decisions are recorded as a 1 response and *reject* decisions are recorded as a 0 response as done previously [16, 19, 20, 27, 42]. Thresholds were calculated separately for each trial using a leave-one-out approach. For trial *i* and category *c*, the Heaviside step function was fed all trials excluding trial *i*; this yielded a vector of thresholds equal to the number of trials for category *c*. We averaged the vector and used the averaged threshold value for remaining analysis.

#### Reward earnings

The number of videos earned (i.e., watched) from each category were tallied. Video categories were then ordinally ranked, within session, based on the number of videos earned in each category. This approach follows from prior studies of mice during Restaurant Row in which ordinal rankings were derived based on the number of pellets earned in each restaurant [19–21, 43]; our primary Web-Surf metric differs from those reported recently in [18] and was an effort to align with the earlier animal studies.

We parsed earning counts based on more preferred (ranks 1 + 2) and less preferred (ranks 3 + 4) and then created a more – less preferred difference score. Higher, more positive scores reflect more successful pursuit of preferred video rewards. Primary analyses considered the difference score, with follow-up analyses probing more and less preferred earnings, separately. Because some testing sessions were shorter, the reward earning variable was normalized by session duration; this allowed us to include data from all sessions in the same model.

In humans, ordinal rankings can also be determined using stated preferences (i.e., video ratings). Supplemental analyses evaluated whether effects were comparable when using rankings derived from video ratings (averaged within category). We also considered whether the results remained when using category-specific delay thresholds to compute ordinal rankings.

#### Hedonic enjoyment

Video ratings (regardless of category) were averaged within each session, and then across baseline sessions (data from Weeks -2, -1, 0 were acquired prior to rTMS). Higher values indicate more overall enjoyment.

#### Depression/anhedonia symptoms

Average PHQ-9 scores were obtained at baseline (Week 0) and end of treatment (Weeks 6 and 7). Depression symptom changes were calculated as end of treatment – baseline. A comparable approach was used to obtain an anhedonia symptom change score, using the PHQ-9 item, which asks if patients have had “little interest or pleasure in doing things.”

### Statistical analyses

To evaluate Web-Surf’s reliability and validity in the MDD population: 1) we examined whether thresholds were stable across sessions, as they were for a non-clinical human sample [18] and rodents [12, 25, 44]. We implemented a linear mixed-effects model: Threshold ~ Category + Week + Category X Week + (Week|Patient); we nested Week within Patient because not all patients completed every testing session. We removed Week -2 from this analysis as only one person completed that session (Fig S1). We similarly tested whether hedonic video ratings were stable across sessions: Rating ~ Category + Week + Category X Week + (Week|Patient). 2) To evaluate construct validity for each session, we correlated the four category-specific delay thresholds with average video ratings from each category [14]; this yields a single value for each participant, for each session. These values reflect the degree of alignment between revealed (i.e., delay thresholds) and stated preferences (i.e., video ratings).

We next evaluated whether reward earnings and hedonic enjoyment differed for patients with MDD, at baseline (prior to rTMS), when compared to a previous community-based sample who completed the task online via the Prolific platform up to three times on different days (*n* = 140) [18]. Here we compared the 1) distributions of video reward earnings (for more – less preferred categories) and 2) average video ratings via independent-sample *t*-tests. We used F-tests to confirm the samples did not differ in variance before implementing the *t*-tests. Intraclass Correlation Coefficients (ICCs) indicated good-to-excellent test-retest reliability of our novel earning metric (Supplemental Materials). To validate the preference-based reward earning metric as a proxy for successful reward pursuit, we correlated video reward earning counts with average video ratings in each sample (MDD, online community participants), separately, using Spearman correlations. Lastly, to assess the construct validity for our Web-Surf metrics, we correlated the preference-based reward earning metric and average video ratings with depression and anhedonia scores in each sample (MDD, online community participants) via Spearman correlations. Online community participants completed several trait/symptom questionnaires to measure depression and anhedonia in the general population, including the Center for Epidemiological Studies – Depression (CES-D [45]) and the Temporal Experience of Pleasure Scale (TEPS [46]). Approximately half of the community participants were at risk for depression based on the CES-D cutoff of ≥ 16; consequently, the online sample is suitable for probing individual differences in key task metrics in a larger sample who received more comprehensive trait/symptom inventories. Additional details on the comparison sample are in Supplemental Materials. We used the false discovery rate (FDR) to adjust *p*-values from related correlation tests and associated follow-up tests.

To examine reward earnings as a predictive biomarker for rTMS response, we correlated baseline video reward earnings (for more – less preferred categories) with symptom changes (end-of-treatment – baseline) via Spearman correlations. This analysis was first conducted using change in total PHQ-9 scores and then using change in the anhedonia item, only. For significant correlations, we performed follow-up tests that controlled for baseline symptom levels using partial Spearman correlations, given that baseline anhedonia was inversely related to anhedonia change scores [47]. A partial Spearman correlation was similarly used to test whether significant effects remained when accounting for demographic and clinical covariates: Age was included given our wide age range among participants; biological Sex was included given a numerically greater though not significant symptom reduction in women (*t*_28_ = -1.77, *p* = .09); Coil (Figure-8, H1) was included to assess whether more focal versus diffuse rTMS treatment targeting influenced behavioral changes. We also considered whether any observed anhedonia results were specific to anhedonia or could be similarly captured by changes in the other core MDD symptom, depressed mood, using the PHQ-9 item asking if patients have been “feeling down, depressed, or hopeless” and Spearman correlation. We used the FDR to correct *p*-values from related correlation tests and associated follow-up tests.

To probe whether reward trajectories could represent a treatment engagement biomarker, our final analysis used a linear mixed-effects models to test whether reward pursuit was enhanced over the rTMS therapy course, particularly for patients who showed reduced anhedonia. We created a binary metric that parsed patients whose anhedonia symptoms declined from baseline to end-of-treatment (*n* = 18) from those who showed no improvement or a worsening of anhedonia (*n* = 7). This binary metric was included in the model as a moderator variable: Number of Videos Earned (More – Less Preferred Categories)/Session Duration ~ Week X Anhedonia Reduction + (Week|Patient). Age, Sex, and Coil type were tested as covariates in a separate mixed-effects model. Additional follow-up models controlled for baseline anhedonia or depression severity. We used the FDR to correct *p*-values from associated follow-up tests.

## Results

### Patients with MDD exhibit stable economic behaviors

Patients with MDD established individual preferences and made economic choices based on those preferences (Fig. 1b, Fig S2). Delay thresholds were stable throughout treatment (Fig. 1c), as indicated by a main effect of Category (*χ*² = 277.79, *p* < .001), but not Week (*p* = .84), and no interaction of Category with Week (*p* = .47). Computation of the Bayes factor indicated that the addition of the Category by Week interaction term was not favored (BF_01_ = 867.91). Video ratings were also stable, evidenced by a non-significant Category X Week interaction (*p* = .11) and a Bayes factor that provided strong evidence against a change in ratings based on Week (BF_01_ = 64.94). Category thresholds correlated with hedonic video ratings throughout the treatment course (average validity correlations above 0.6 for every week; Fig. 1d).

### People who earn more relatively more preferred rewards report greater hedonic pleasure

**Fig. 2:**
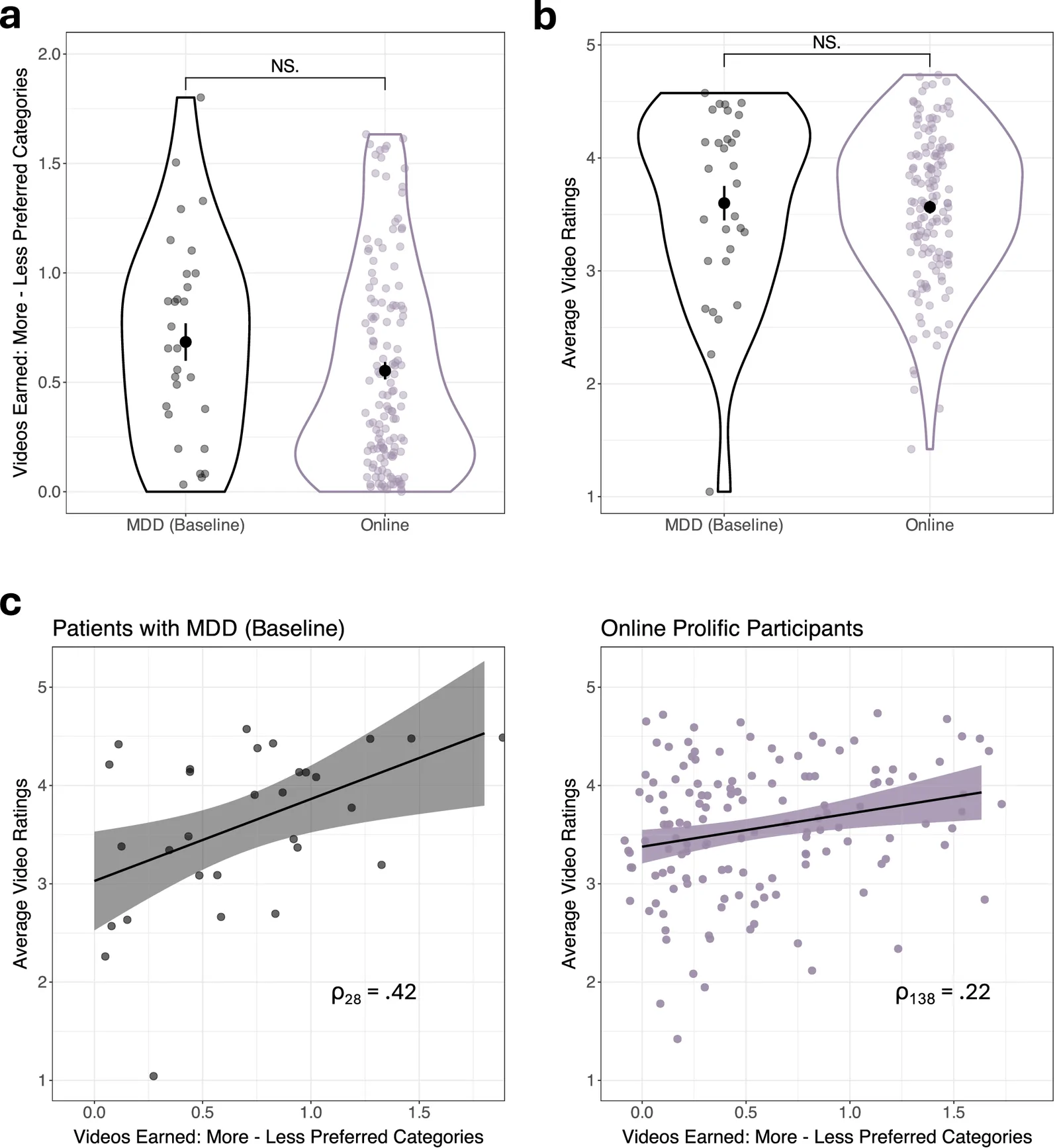
Relations between video earnings and hedonic enjoyment measured from Web-Surf. At baseline, before repetitive transcranial magnetic stimulation therapy. **a** distributions of video earnings and **b** overall hedonic enjoyment were similar among patients with major depressive disorder (MDD) and community participants who completed Web-Surf as part of an online study on the Prolific platform (Online). **c** The tendency to earn more preferred and fewer less preferred video rewards correlated with greater hedonic enjoyment. Abbreviations: MDD major depressive disorder.

At baseline, patients with MDD and community participants did not differ on average, for reward earnings counts (more – less preferred) (*t*_168_ = -1.41, *p* = .16; Fig. 2a) or hedonic enjoyment (*t*_168_ = -.26, *p* = .79; Fig. 2b). Furthermore, reward earnings (more – less preferred) positively correlated with hedonic video ratings in patients with MDD (ρ_28_ = .42, *p* = .02) and community participants (ρ_138_ = .22, *p* = .008) (Fig. 2c). This was driven by a positive association between more preferred earnings and ratings (ρ_28_MDD_ = .30, *p* = .10; ρ_138_Online_ = .21, *p* = .01, *p*_adj_ = .03) and a negative association between less preferred earnings and ratings (ρ_28_MDD_ = -.46, *p* = .01, *p*_adj_ = .03; ρ_138_Online_ = -.10, *p* = .22). Essentially, people earning more preferred and fewer less preferred videos reported more hedonic pleasure, though not all follow-up within-condition slopes reached significance. Better delineation between reward categories, i.e., exhibiting clearer preferences, may therefore be the driving factor. This notion is verified by tight correlations between reward earning counts (more – less preferred) and the standard deviation of category-specific delay thresholds (ρ_28_MDD_ = .92, *p* < .001; ρ_138_Online_ = .80, *p* < .001). Comparable findings were seen when ordinal preferences were derived from delay thresholds or ratings.

Baseline reward earnings (more – less preferred) and hedonic enjoyment were unrelated to baseline PHQ-9 depression (both *ps* > .70) and anhedonia scores (both *ps* > .70) among patients with MDD. In the online community sample (Fig S3), reward earnings (more – less preferred) negatively correlated with CES-D depression scores (ρ_70_ = -.32, *p* = .006, *p*_adj_ = .02) and positively correlated with TEPS consummatory pleasure ratings (ρ_70_ = .24, *p* = .04, *p*_adj_ = .07), but not TEPS anticipatory pleasure ratings (*p* = .56). This suggests that community participants who earned relatively more preferred and fewer non-preferred rewards endorsed less depression and more enjoyment from events in everyday life (though we caution that the consummatory pleasure association did not survive multiple comparison correction). Hedonic video ratings correlated with anticipatory pleasure ratings (ρ_70_ = 0.29, *p* = .01, *p*_adj_ = .03) and consummatory pleasure ratings (ρ_70_ = 0.27, *p* = .02, *p*_adj_ = .03) (Fig S3), but were unrelated to depression scores (*p* = .42). That is, community participants reporting greater hedonic enjoyment also endorsed more pleasure from events in everyday life. All significant effects were similarly observed when ordinal category preferences were based on delay thresholds or ratings. Additional specificity analyses are provided in Supplemental Materials.

### Patients with MDD who earn more preferred rewards at baseline show greater anhedonia reductions following rTMS

**Fig. 3:**
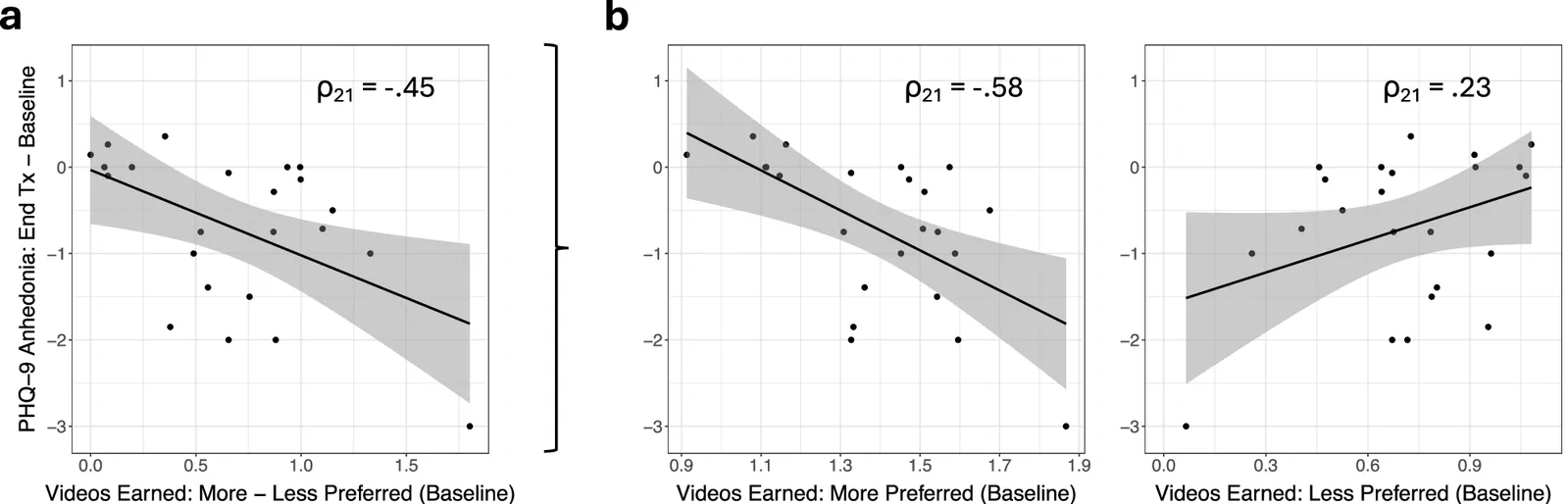
Baseline reward earning patterns correlate with anhedonia reductions following repetitive transcranial magnetic stimulation (rTMS). At baseline, before repetitive transcranial magnetic stimulation therapy, patients with major depressive disorder who earned relatively more preferred rewards showed **a** greater reductions in anhedonia symptoms. **b** Anhedonia reductions specifically correlated with greater earnings of more preferred rewards at baseline (*left*) and were unrelated to less preferred earnings (*right*). Note that video earnings were normalized based on the task duration for that respective testing session. Abbreviations: PHQ-9 Patient Health Questionnaire, Tx treatment.

Baseline reward earnings (more – less preferred) did not significantly correlate with total PHQ-9 change scores (ρ_21_ = -.35, *p* = .10), or anhedonia change scores, after FDR correction (ρ_21_ = -.45, *p* = .01, *p*_adj_ = .06; Fig. 3a). There was, however, a correlation of the anhedonia change score with more preferred earnings (ρ_21_ = -.58, *p* = .003, *p*_adj_ = .007), but not less preferred earnings (ρ_21_ = .23, *p* = .28; Fig. 3b). This suggests that patients with MDD earning more preferred video rewards prior to rTMS showed the largest drops in anhedonia. When ordinal rankings were based on category-specific thresholds, we observed similar associations between anhedonia change scores with baseline earnings for more – less preferred rewards (*p* < .05) and with baseline earnings for more preferred rewards (*p* < .01); these associations were absent for rankings based on video ratings. Additional analyses accounting for clinical, demographic, and treatment covariates, or when restricting the analyses to sessions at least 20 min long, further suggest that baseline earnings of more preferred rewards more strongly correlated with anhedonia reductions than the earning difference score (Supplemental Materials).

We found a non-significant association between baseline reward earnings (more – less preferred) and mood symptom changes (ρ_21_ = -.16, *p* = .46) based on the respective PHQ-9 item; the equivalent Spearman correlation using baseline earnings from more preferred categories was also not significant (*p* = .09).

### Over the rTMS course, patients with MDD increase their relative earnings of more preferred rewards

Anhedonia reductions moderated earning trajectories over the rTMS course. The Week X Anhedonia Reduction interaction was significant (*χ*² = 4.65, *p* = .03; Fig. 3b), and was driven by a positive increase in more – less preferred video reward earnings among patients with reduced anhedonia scores (*t*-ratio_22_ = 2.87, *p* = .009; *p*_adj_ = .02); no behavioral change was observed in patients with anhedonia scores that did not improve or worsened (*t*-ratio_22_ = -0.81, *p* = .43). Similar findings were observed when ordinal preferences were based on category-specific thresholds or ratings (although attenuated to *p* = .13 for rating-based preference rankings), when accounting for clinical, demographic, and treatment covariates, and when restricting the analyses to sessions at least 20 min long (Supplemental Materials).

In follow-up analyses we separately tested whether earning trajectories for more and less preferred categories were modulated by changes in anhedonia following rTMS. The interaction term for the model of more preferred earnings was not significant (*χ²* = 2.85, *p* = .09). After dropping the interaction term, we attained a significant main effect of Week (*χ²* = 7.86, *p* = .005) characterized by a positive slope, indicating an increase in more preferred earnings over time regardless of anhedonia-change status. We observed a significant interaction for the model of less preferred earnings (*χ²* = 5.44, *p* = .02, *p*_adj_ = .04); unpacking this interaction revealed a modest decline in less preferred earnings among patients with reduced anhedonia after rTMS (*t*-ratio_22_ = -2.34, *p* = .03, *p*_adj_ =.06) that did not survive FDR correction. Patients who did not improve or got worse showed no change in less preferred earnings (*p* = .20). Taken together, better reward pursuit optimization was observed among patients for whom anhedonia levels were reduced with rTMS, and this slope difference was more robust when quantified with the reward earning difference score. Additional supplemental models examine trends within each category rank (Figure S4), and beyond the 7-week cutoff we used from the primary analyses (Figure S5).

## Discussion

**Fig. 4:**
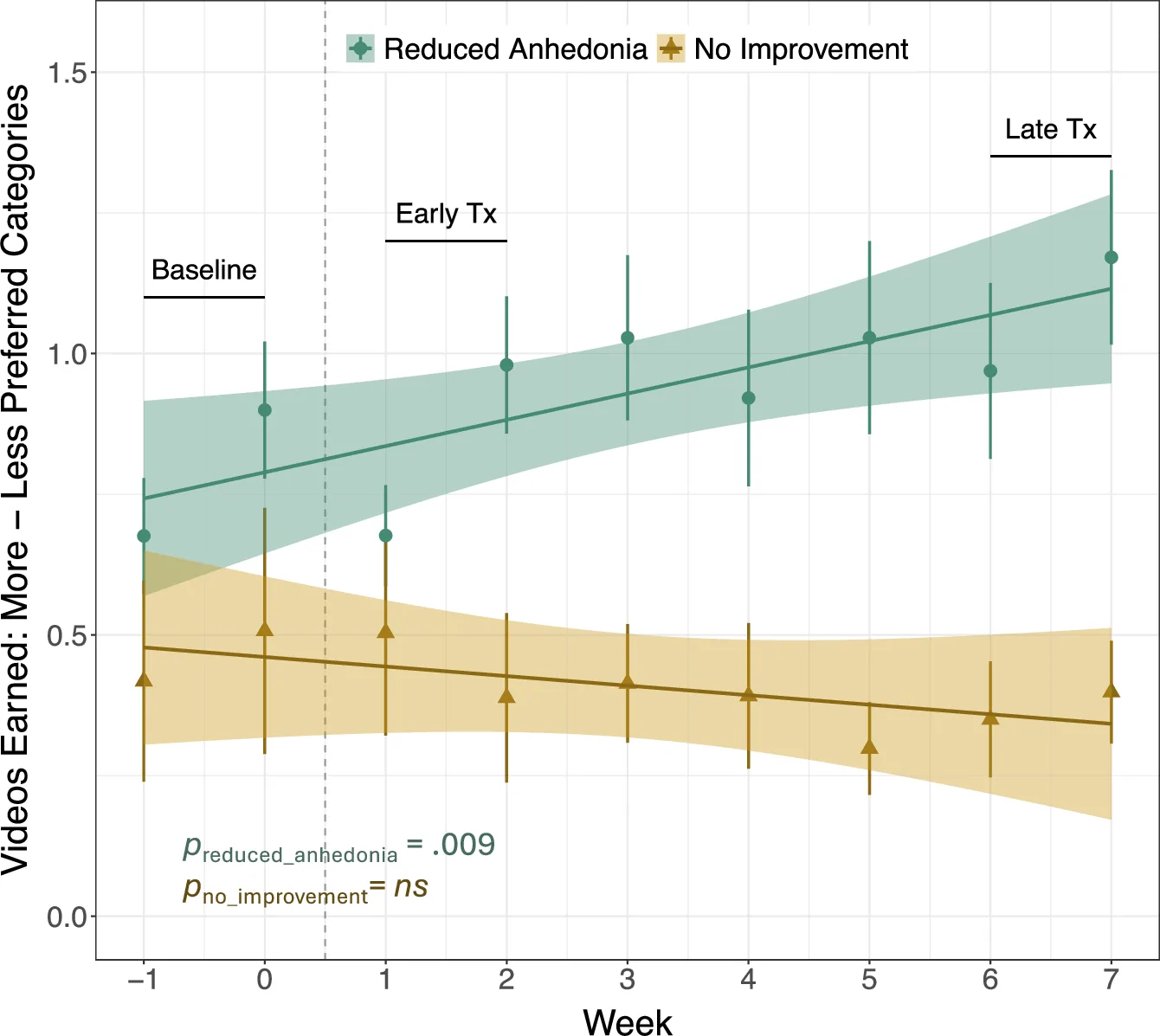
Changes in reward earnings over the course of repetitive transcranial magnetic stimulation (rTMS) therapy depend on treatment response. Patients with major depressive disorder increased their relative earnings of more preferred versus less preferred video rewards, particularly those with reduced anhedonia symptoms following repetitive transcranial magnetic stimulation (rTMS). Conversely, patients whose anhedonia symptoms did not improve (or worsened) showed no change in reward pursuit patterns over time. Data shown as the mean ± standard error of the mean across participants. Note that video earnings were normalized based on the task duration for that respective testing session. Abbreviations: Tx treatment.

We show that a translational marker of reward pursuit correlates with anhedonia reductions following rTMS in patients with MDD. During a foraging task with real-time delays and primary rewards (Web-Surf), humans make accept/reject decisions to pursue video rewards from four categories (kittens, dance, landscapes, bike accidents). Successful reward pursuit in community participants (based on video earnings) correlated with lower depression symptoms and greater pleasure ability (though the latter effect did not survive multiple comparison correction). Hedonic video ratings among community participants correlated with pleasure ability but not depression symptoms. The rate of earning for more versus less preferred rewards increased over repeated testing sessions, particularly for patients whose anhedonia symptoms declined with rTMS therapy. Patients earning more preferred rewards, at baseline, had the largest reductions in anhedonia. Our results were generally robust to how category preferences were defined (albeit weaker when preferences were based on hedonic ratings) (Fig. 4).

Like mice [19, 20], patients with MDD learned to increase their relative earnings of more preferred video rewards over the rTMS course. Changes in reward pursuit were most evident for patients who declined in anhedonia symptom severity. This aligns with evidence from mice who experienced chronic social defeat, a model for inducing depression-like phenotypes [48]. Stress-resilient mice were more efficient at reward optimization in a manner that prioritized their flavor preferences [19]. In the context of depression, one possibility is that patients with alleviated symptoms are better able to represent their desired future outcomes. When making decisions during Restaurant Row, an animal’s brain shows representations of the future outcome [12]. Neural representations measured with functional magnetic resonance imaging (fMRI) similarly show future and competing options preceding reject decisions during Web-Surf [27]. Future thinking involves imagining and evaluating potential outcomes that help to facilitate anticipatory pleasure [49]. Vivid mental imagery can emulate real experiences and emotional responses [50]. Pessimistic future-thinking is a hallmark depression symptom [51, 52]. People with MDD often struggle to generate detailed representations of possible futures [53], particularly positive events [54–56], which may reduce anticipatory pleasure and motivation to seek out rewards [57, 58]. Aberrant mental simulation may impede reward pursuit at the choice point in MDD, particularly in patients who remained symptomatic after treatment. This hypothesis is supported by evidence that people with depression rely less on rich, internal task representations during exploratory decision-making [59]. Relatedly, cognitive capacity may influence successful reward pursuit in MDD. The theory of “policy compression” captures how action selection depends on an individual’s capacity limit: when capacity is low, the individual is state-insensitive and more likely to pursue generic items; whereas, when capacity is high, the individual is highly state-dependent and can make more complex decisions to pursue premium rewards [60]. Policies of high capacity require greater memory to implement. In line with policy compression, people with MDD exhibit reduced working memory and increased perseverative thinking and errors [61–63]. Future work is needed to clarify how basic memory, learning, and prospection processes support reward pursuit during Web-Surf and whether those relationships differ based on depression symptomatology.

Reward pursuit changes following rTMS may relate to putative biomarkers of treatment response, potentially involving key depression-related structures such as subgenual anterior cingulate cortex (sgACC) and nucleus accumbens (NAcc) [39, 64, 65]. Connectivity between the prefrontal cortex and sgACC is linked with better clinical response to rTMS [66–69], although in the largest dataset to date, connectivity between the sgACC and prefrontal stimulation site only explained a small proportion of variance in treatment outcomes [70] and there are mixed findings as to whether more positive versus negative connectivity between these regions is optimal [64]. rTMS may also normalize aspects of aberrant sgACC connectivity in people with depression [71]. Greater negative connectivity between the dorsolateral prefrontal cortex-to-NAcc has been linked with antidepressant response [65], while aberrant NAcc connectivity corresponds with anhedonia severity in people with depression [72, 73]. Decisions during Web-Surf recruit the anterior cingulate cortex and video viewing (i.e., reward consumption) recruits the NAcc [27]. This raises interesting hypotheses as to whether baseline sgACC and NAcc connectivity levels can predict reward pursuit changes with rTMS, or if rTMS is improving connectivity in circuits relevant to both depression treatment response and successful reward pursuit. It is worth noting that our rTMS approach differed from these prior fMRI studies in that it lacked neuronavigation-guided targeting, and there was notable treatment heterogeneity across patients that could influence which underlying brain regions were modulated [74].

Patients with MDD who earned more preferred rewards on Web-Surf, prior to rTMS, showed larger anhedonia reductions following rTMS. Baseline earnings (more – less preferred categories) also correlated with baseline video ratings, i.e., patients and community participants who earned relatively more preferred rewards reported a more pleasurable experience overall. Conceptually, this indicates an adaptive coherence between the distinct motivational components of “wanting” (incentive salience reflected in the motivational drive to wait for and earn a video reward) and “liking” (pleasure obtained from viewing that video reward) [75, 76]. Conversely, people who watched a similar number of videos from all categories, i.e., did not vary in how much they preferred some reward types over others, also reported less enjoyment on average, and were less responsive to rTMS. For some people with MDD, the brain circuity supporting motivated reward pursuit may be intact but perhaps slowed down, such as the case of mood reactivity in atypical depression where a person’s mood temporarily brightens in response to positive events [77]. Improvement from rTMS may be more feasible in people with preserved reward circuitry [78, 79]. That is, it may be easier to reignite a functioning circuit than to repair it. Web-Surf performance could potentially serve as a screening tool for predicting treatment response, though we caution that some of our associated correlation findings were modest and did not survive multiple-comparison correction. Task-derived metrics could alternatively be used for treatment target selection. For example, compared to treatment responders, individuals who do not show an antidepressant response to dorsomedial prefrontal cortex (PFC)-targeted rTMS report higher levels of anhedonia and exhibit disrupted connectivity in medial PFC-connected reward circuitry [78]. Here we observed intact reward circuit function in individuals who improved in anhedonia symptoms after rTMS. Subregions of the PFC may form partially dissociable circuits which when targeted with rTMS, provide separable benefits on specific dimensions of MDD symptomatology. Specifically, lateral PFC targets are associated with frontoparietal control circuits which impart benefit on dysphoric symptoms of MDD, whereas medial PFC targets show stronger coupling with limbic and salience networks associated with anxiosomatic dimensions of MDD [80]. Task-derived metrics might therefore inform individualized targeting strategies based on specific manifest circuit deficits; an approach that has been proposed but not yet prospectively validated. This general approach also highlights the precise goals of the Web-Surf and Restaurant Row cross-species endeavor, through which we seek to forward and back translate findings to study the validity of circuit-specific computational processes [10], and to examine processing algorithms that cut across diagnoses or along transdiagnostic symptom dimensions [43, 81, 82].

These findings have potential implications for depression-focused cognitive behavioral therapy. Behavioral activation asks people with depression to create an activity schedule to facilitate positive reinforcement from the environment that in turn, can facilitate more motivation [83]; this activity ideally fosters synergy between those wanting and liking mechanisms; and as alluded to above, individual differences in this synergy can be measured with Web-Surf.

Because we are the first to study depression with Web-Surf, we re-examined the basic psychometric properties of Web-Surf in this sample. Patients with MDD made reliable, value-based decisions. Like prior human samples [14], self-reported enjoyment was generally higher after watching videos they were willing to wait longer for. Category-specific delay thresholds and video ratings were stable across repeated testing as expected based on prior work [18]. Overall hedonic experience, at baseline, was no different for patients with MDD compared to non-psychiatric, community-based participants. In support of Web-Surf’s construct validity (specifically its experiential rewards), hedonic enjoyment among community participants during Web-Surf maps on to self-reported hedonic ability in everyday life. Web-Surf performance revealed aspects of intact reward processing for immediately consumable rewards; this aligns with evidence that approach motivation is often intact or even enhanced in depression in the context of primary reward [84, 85]. While baseline earnings did not directly correlate with baseline depression symptoms or anhedonia levels in patients with MDD, community participants who earned more of their preferred rewards exhibited lower depression symptoms and greater pleasure ability. This provides a direct link between accept/reject computational processes and anhedonia. Differences in the presence of these correlations across samples could be due to a number of factors. First and foremost, the PHQ-9 is a screening and diagnostic tool that may not capture anhedonia as adequately as anhedonia-specific instruments, like the TEPS. The PHQ-9 may also lack the nuance of more comprehensive inventories, like the TEPS and CES-D. The samples also differed in basic demographics (race, sex) and task administration style (in-person, remote). Patients with MDD were recruited from an academic psychiatry clinic as part of a naturalistic, observational study; eligible participants included people in Minnesota who were referred for rTMS depression treatment. This approach is notably different from the Prolific platform that recruited people from all over the United States (and the demographics of the broader United States are inherently different from those of suburban Minnesota). Patients with MDD also completed the tasks in person. The online participants were not in person, although the Prolific platform included a built-in monitoring system to check task engagement. This proctoring differed, however, from the proctoring used in the MDD sample (where the screen and webcam were actively monitoring by a human). Our MDD sample size may also be insufficient to observe these effects. Based on an assumed power of .80 and significance level of .05, power analyses revealed that an estimated sample size of 73 – 133 participants is needed to obtain the small-to-medium effect sizes (Spearman correlations ranging .24 to .32) we observed in the online sample. We hope this can provide a benchmark for future studies.

Several limitations should be acknowledged. Our MDD sample was small. Given a sample size of approximately 30 patients, it is possible that our repeated-measures models containing more than two predictors may have unstable estimates (although our linear mixed-effects models benefited from most participants having at least 7 timepoints). We lacked a non-treatment comparison group. We therefore cannot rule out the possibility that the observed improvements in reward-seeking are not due to more general treatment factors that are not specific to rTMS therapy. The study was observational, and patients were not randomized; instead, treating clinicians determined the optimal treatment protocol for each patient. This approach lacks certain experimental controls but has inherent strengths in its generalizability to the clinic. Many patients were taking a variety of medication types (e.g., antianxiety, anticonvulsant, antidepressant). While it is possible that medication heterogeneity could influence treatment outcomes, the current study was not sufficiently powered to examine the contributions of medication status to the observed changes in reward pursuit. Future studies are needed to understand whether reward pursuit changes are broadly related to prefrontal augmentation (H1 coil), more specific to dlPFC function (F8 coil), or are simply due to clinical improvement irrespective of neural targeting. The inclusion of fMRI would further elucidate whether rTMS-related changes are due to prefrontal cortex modulation and clarify whether treatment response depends on the inherent integrity of one’s reward circuitry. Only one clinical measure (PHQ-9) was collected in patients with MDD. The PHQ-9 is a widely used screening tool for assessing depression severity; it was not designed to study anhedonia, specifically. The anhedonia item from the PHQ-9 may also conflate different components of anhedonia (i.e., loss of pleasure and loss of interest). Anhedonia is a multi-faceted construct that parses into anticipatory and consummatory components. Based on evidence in community participants, some anhedonia facets may relate to reward pursuit more so than others (although effects were generally small). Future studies should include a more comprehensive set of anhedonia and depression instruments to clarify how reward-pursuit metrics differentially relate to specific facets of depression. More comprehensive instruments can clarify whether specific anhedonia facets respond most to rTMS therapy. Larger trials with appropriate statistical power are better suited to address questions about individual differences in clinical response based on Web-Surf decision parameters. Follow-up studies can also clarify how motivation based on willingness-to-wait (during Web-Surf) compares with effort-based foraging tasks (like [86, 87]). Relatedly, it is important to disentangle how reward pursuit during Web-Surf relates executive function versus pleasure ability (i.e., anhedonia). Findings in community participants revealed that executive dysfunction, measured with a trait impulsivity questionnaire, correlated with poorer reward pursuit more generally, whereas pleasure ability specifically correlated with successful attainment of more preferred rewards (Supplemental Materials).

In conclusion, we used a rodent-to-human foraging task to capture value-based reward pursuit in patients with MDD who underwent standard rTMS therapy. We demonstrate a novel, translational reward pursuit metric that may improve among patients who show a clinical response. Our findings highlight the value for identifying specific decision-making processes in depression as novel treatment targets [88]. This work also lays the groundwork for larger-scale trials seeking to clarify the specific mechanisms that underlie motivational impairments in MDD, such as memory and imagery processes involved in reward pursuit.

## Supplementary information

Supplemental Material

## Data Availability

Data will be made available upon request.
