# Supplementary material for: Reward pursuit during a translational reward task correlates with anhedonia reductions following rTMS in patients with major depressive disorder
Source: Transl Psychiatry. 2026 Jul 10;16:514. doi: 10.1038/s41398-026-04248-3 (PMC13635226; doi:10.1038/s41398-026-04248-3)
Supplement: Supplementary file 1 [file 41398_2026_4248_MOESM1_ESM.pdf]

Supplemental Materials

Reward pursuit during a translational foraging task correlates with anhedonia reductions following rTMS in patients with major depressive disorder

Samantha V. Abram, Aaron N. McInnes, Christi R.P. Sullivan, Dawson C. Cooper, Brian M. Sweis, Alik S. Widge

Additional sample characteristics
Major Depressive Disorder (MDD) Sample: Thirty-two individuals with treatment-resistant MDD completed this study (ages 20 to 84 years old, mean age = 50.49 ± 17.29; 18 women/14 men; 100% Caucasian). Participants were recruited from an academic interventional psychiatry clinic in suburban Minnesota and required to be at least 18 years of age. This study used a naturalistic, observational design in which participants completed study procedures in parallel with their standard clinical care. This study did not include randomization.

Eligible participants were those scheduled or on waitlist to begin a clinically indicated course of outpatient repetitive transcranial magnetic stimulation (rTMS) for MDD at the recruiting treatment-resistant depression neuromodulation clinic. Participants were seeking treatment for a primary diagnosis of MDD, although many had comorbid diagnoses (Table S1). Eligible participants were identified by medical record review and were contacted by the study team for enrollment. Treatment decisions, including treatment eligibility criteria and stimulation parameters, were determined by the treating clinical team and were not influenced by study participation. Eligibility for a clinical course of rTMS was based on FDA-approved indications, defined by persistent depressive symptoms despite prior antidepressant treatment in the current episode. Augmentation strategies in combination with primary monoaminergic treatment were encouraged but not required, as was participation in evidence-based psychotherapy. Adequacy of prior medication trials was verified by medical record review as part of the standard clinical care, as were MDD and comorbid diagnoses. Medication data are provided in Table S2. We assessed the level of treatment resistance of the sample by medical record review of clinician ratings from the antidepressant treatment history form (ATHF; Sackeim et al., 2019). ATHF data were unavailable for 8 participants. For the remaining 24 participants, ratings of adequacy and duration of prior antidepressant trials ranged from 3 – 18, with a mean of 9. No minimum threshold of depression severity was required for treatment eligibility. No individuals with a diagnosis of bipolar disorder participated in the study, which was confirmed by medical record review.

Online Community Sample: Two-hundred eighty-four individuals recruited from the online platform Prolific completed Web-Surf either once (*Single*, n = 145; ages 18 to 78 years old, mean age = 36.88 ± 12.11, 78 men/67 women; 70% Caucasian, 11% Black, 5% Asian, 6% mixed race, 6% other) or up to three times (*Repeat*, n = 140; ages 19 to 72 years old, mean age = 36.78 ± 13.24; 72 male/65 female/1 prefer not to say; 64% Caucasian, 12% Black, 8% Asian, 6% mixed race, 7% other) as part of a study on task reliability (Mcinnes, Sullivan, MacDonald III, & Widge, 2025). Participants were required to be at least 18 years of age and living in the USA. Analyses for the current paper included only Prolific participants who participated in the repeated protocol.

All procedures for the online Prolific study were approved by the Institutional Review Board at the University of Minnesota (STUDY00007274) and comply with the ethical standards of the Declaration of Helsinki. Informed consent was obtained from all participants in accordance with the Declaration of Helsinki. Participant data is fully anonymized and not to be identified from the manuscript.

rTMS Administration (MDD Sample)
Patients received rTMS of the dorsolateral prefrontal cortex five days per week, with 36 treatments in a full series, with a Figure-8 (n = 21) or H1 (n = 11) coil. Both the more precise Figure-8 coil and the relatively diffuse H1 coil are efficacious for reducing depression and anhedonia symptoms (Sonmez et al., 2024). Within a given session, the Figure-8 protocol targeted F3 (left hemisphere), F4 (right hemisphere), or both (bilateral) (Table S3), using the Beam-F3 targeting method (Beam, Borckardt, Reeves, & George, 2009). The H1 coil was centered over the eye or eyebrow areas. Figure-8

treatments were delivered at a frequency of 1, 5, 10, 33, or 50 (intermittent theta burst) Hz; H1 treatment was delivered at an 18 Hz frequency (Table S4). On occasion, the rTMS protocol was changed during the treatment course at the clinician discretion. The specific protocol employed was determined by the treating clinician, hence the variation within and across patients. No major adverse events were reported. Some patients indicated mild intolerance of stimulation.

While our primary analyses were restricted to Web-Surf sessions from the first 7 weeks of rTMS, we note that several patients continued rTMS beyond 7 weeks. Table S5 details the number of patients who underwent rTMS each week; these *ns* also correspond to the number of available Web-Surf sessions for data analyses at each timepoint.

Additional task and study variable details

<u>Web-Surf Task</u>: Participants had a fixed amount of time to cycle between four video categories, each providing a different "flavor" of video (i.e., kittens, dance, landscapes, bike accidents). Categories were indicated by a symbol and text (Fig 1a). Categories were presented in a fixed order within a session. It was possible that patients with MDD saw the same video more than once depending on the number of sessions (and trials) they completed. A blue download bar showed the offer in seconds (i.e., delay). If the participant chose to *accept*, the delay and blue bar counted down, the video played for approximately 4 seconds, and the participant rated the video on a 1 – 5 star-rating scale (1 star = extremely disliked, 5 stars = extremely liked). At any point during the delay, the participant could elect to *quit* and end the trial early; in this case they would no longer see the video and would advance towards the subsequent category. Participants also had the option to *reject* an offer and immediately advance towards the next category. Regardless of the decision to accept/reject/quit, participants passed through a travel zone in which they waited 3 seconds before advancing to the next offer (i.e., next category with a new, random delay).

For the MDD sample, Web-Surf was collected weekly as part of a broader cognitive task battery, and task order varied by week. Task duration was fixed at 30 minutes for the online sample (Mcinnes et al., 2025). For the MDD sample, session durations ranged from approximately 4 to 40 minutes, with 99% at least 10 minutes long and 90% at least 15 minutes long.

The task was written in Javascript and hosted on the University of Minnesota's servers. A dedicated laptop in the clinic was used to run the task following treatment sessions. MDD patients completed Web-Surf in-person using a dedicated laptop in the treating clinic; patients were remotely proctored via Zoom software. The online sample was required to use a desktop or laptop computer with audio capabilities when completing Web-Surf. Because Online participants were remote, the Prolific platform included a built-in monitoring system that checked if participants had minimized the browser or switched tabs and consequently quit the program; this approach was to ensure data validity (Mcinnes et al., 2025).

<u>Test-retest reliability of video earnings</u>: Intraclass Correlation Coefficients (ICCs) quantified the test-retest reliability of our novel reward earning (more – less preferred) variable. We based our ICC formula on calculations provided in (Koo & Li, 2016). This analysis revealed excellent reliability among patients with MDD: ICC [95% CI] = 0.93 [0.88, 0.96] and good reliability among online community participants: ICC [95% CI] = 0.89 [0.83, 0.92].

Additional results and discussion

<u>Depression symptom improvement following repetitive transcranial magnetic stimulation (rTMS) in patients with treatment-resistant MDD</u>: The Patient Health Questionnaire (PHQ-9) was used to

capture depression symptom severity throughout treatment, and was completed weekly (Kroenke, Spitzer, & Williams, 2001). Prior to treatment (Week 0), patients scored an average of 14.80 ± 5.90, indicating moderately severe levels of depression. Scores dropped to an average of 11.15 ± 6.99 at the end of treatment (Weeks to 6 and 7), indicating moderate depression levels. This change was characterized by a large effect size (d = .80). A mixed-effects model regressed PHQ-9 scores on Week, with Patient as a random effect, and Week as a nested variable within Patient. This model similarly captured the downward trend in depression symptoms following rTMS treatment ($\chi^2 = 27.35$, $p < .001$; Fig S1).

Depression and anhedonia correlations in online community participants: The Center for Epidemiological Studies – Depression (CES-D; Radloff, 1977) and Temporal Experience of Pleasure Scale (TEPS; Gard, Gard, Kring, & John, 2006) were used to measure depression and anhedonia. These measures are intended for research in general (non-psychiatric) populations. The TEPS parses into two components that capture anticipatory and consummatory pleasure. The anticipatory component includes items such as: "*When I think about eating my favorite food, I can taste how good it is*", and "*I look forward to a lot of things in my life*" The consummatory component includes items such as: *The smell of fresh grass is enjoyable to me*", "*I really enjoy the feeling of a good yawn.*" Questionnaire data was not available for all participants; degrees of freedom are included in the results to indicate the number of available datapoints. Distributions of these measures were as follows: CES-D (16.39 ± 11.24), TEPS anticipatory pleasure (42.93 ± 7.80), TEPS consummatory pleasure (36.35 ± 6.24).

We used Spearman correlations for analyses that assessed whether our primary Web-Surf metrics, i.e., reward earnings (more – less preferred) and hedonic video ratings, were related to depression symptoms and pleasure ability. We used the false discovery rate (FDR) to correct for multiple comparisons across families of correlations, i.e., 1) reward earnings (more – less preferred) with total CES-D, TEPS anticipatory pleasure, and TEPS consummatory pleasure (three tests), and 2) between hedonic video ratings and the same trait/symptom scores (three tests). An FDR correction was also applied to any related follow-up tests.

Reward earnings (more – less preferred) negatively correlated with depression scores ($\rho_{70} = -.32$, p = .006, p_{adj} = .02; Fig S3); this effect was driven by a negative association with more preferred earnings ($\rho_{70} = -.34$, p = .003, p_{adj} = .006) and no relation for less preferred earnings (p = .96). With respect to anhedonia, reward earnings positively correlated with TEPS consummatory pleasure ratings (ρ_{70} = .24, p = .04, p_{adj} = .07; Fig S3), but not TEPS anticipatory pleasure (ρ_{70} = .07, p = .56). Like depression symptoms, the consummatory pleasure effect was driven by a positive correlation between more preferred earnings and TEPS consummatory scores (ρ_{70} = .35, p = .003, p_{adj} = .006) and no association for less preferred earnings (p = .97). This suggests that community participants who successfully sought and experienced enjoyable video rewards also endorsed less depression and more enjoyment from events in everyday life (though we caution that the consummatory pleasure and earning difference score correlation was not significant after FDR correction). Comparable results were obtained when ordinal ranks were based on delay thresholds or video ratings.

Hedonic video ratings were unrelated to depression scores (p = .42). Hedonic video ratings correlated with anticipatory pleasure ratings (ρ_{70}= 0.29, p = .01, p_{adj} = .03) and consummatory pleasure (ρ_{70}= 0.27, p = .02, p_{adj} = .03). That is, community participants reporting greater hedonic enjoyment also self-reported more expected and experienced pleasure from events in everyday life.

Here we observe a possible dissociation in how depression symptoms and anhedonia relate to Web-Surf performance: only pleasure ability correlated with hedonic enjoyment, while both pleasure ability and depression were related to successful reward pursuit.

Trait impulsivity correlations in online community participants: We also considered whether other aspects of executive function are relevant to successful reward pursuit during Web-Surf. To this end, we probed trait impulsivity associations using the Barratt Impulsivity Scale (BIS-11; Patton, Stanford, & Barratt, 1995). The BIS-11 parses into three components that capture attentional impulsivity, motor impulsivity, and non-planning impulsivity. Questionnaire data was not available for all participants; degrees of freedom are included in the results to indicate the number of available datapoints. For the BIS-11, participants scored and average of 59.35 $\pm$ 12.01.

When using total BIS-11 scores, we observed a negative association between reward earnings (more – less preferred) and trait impulsivity (ρ_{70}= -.39, p < .001); comparable associations were seen when using each of the individual BIS-11 sub-facets. Unpacking this correlation revealed that higher trait impulsivity was linked with both fewer earnings of more preferred rewards (ρ_{70}= -.30, p = .01, p_{adj} = .02) and more earnings of less preferred rewards (ρ_{70}= .26, p = .02, p_{adj} = .02). This pattern differs from depression and anhedonia which were solely related to more preferred earnings. It further suggests a dissociation between how executive dysfunction and depression/anhedonia are captured in Web-Surf: that is, pleasure ability uniquely scales with obtaining more enjoyable rewards (and therefore can capture approach motivation deficits common to depression (Nusslock, Mittal, & Alloy, 2025)), while impulsivity may underlie poor reward pursuit more broadly.

Baseline earnings from more preferred categories correlate with anhedonia reductions when accounting for clinical, demographic, and treatment covariates, as well as experimental constraints: Partial Spearman correlations showed that baseline earnings (more – less preferred) and rTMS-induced anhedonia reductions remained modestly correlated when accounting for baseline anhedonia (ρ_{21} = -.42, p = .05, p_{adj} = .05) or depression (ρ_{21} = -.46, p = .03, p_{adj} = .05) severity, though these effects were attenuated to non-significance with FDR correction. Like the primary analyses, these correlations were significant with correction when restricted to earnings from more preferred categories (controlling for baseline anhedonia: ρ_{21} = -.56, p = .007, p_{adj} = .007; controlling for baseline depression: ρ_{21} = -.60, p = .003, p_{adj} = .006). Similarly, when controlling for Age, Sex, and Coil, earnings (more – less preferred) did not significantly correlate with anhedonia reductions (ρ_{21} = -.41, p = .07, p_{adj} = .07) but earnings for more preferred videos did correlate with anhedonia reductions (ρ_{21} = -.62, p = .004, p_{adj} = .007).

We also considered whether the level of treatment resistance impacted associations between baseline earnings and anhedonia reductions, as the degree of treatment resistance can predict treatment outcomes. Information on prior psychotropic medication trials was available for a subset of participants. Partial Spearman correlations showed that baseline earnings (more – less preferred) correlated with rTMS-induced anhedonia reductions (ρ_{17} = -.62, p = .006, p_{adj} = .01), but not with total depression symptoms (p = .26), when accounting for the number of prior antidepressant trials. The number of prior antidepressant trials did not directly correlate with anhedonia or total depression symptom reductions (both *ps* > .15).

Due to clinic scheduling constraints, some Web-Surf sessions had shorter durations (~10 – 20 minutes). Consequently, we tested whether our correlation effects remained when restricting analyses to include only baseline sessions at least 20 minutes long. Like the analyses above, the correlation

between baseline earnings (more – less preferred) was not significant (ρ_{19} = -.42, p = .06), while the correlation for only more preferred earnings was indeed significant (ρ_{19} = -.54, p = .01, p_{adj} = .02).

Reward earning trajectories when accounting for clinical, demographic, and treatment covariates, as well as experimental constraints: In the model examining reward earning trajectories (more – less preferred), Age, Sex, and Coil were not significant predictors (all ps > .50), and did not undermine the Week X Anhedonia Reduction (p = .03) or positive slope among patients who showed anhedonia reductions (p = .009, p_{adj} = .02). This pattern of results also remained when accounting for baseline anhedonia levels or depression symptom severity (both interaction ps < .05), with follow-up tests showing comparable reward earning increases among patients who showed rTMS-induced anhedonia reductions (both ps = .009).

We also tested whether our earning trajectory results were significant when including only sessions at least 20 minutes long. We observed a significant Week X Anhedonia Reduction interaction (χ^2 = 4.06, p = .04) driven by an increase in more – less preferred reward earnings among patients who showed rTMS-induced anhedonia reductions (t-ratio_{19}: 2.41, p = .03, p_{adj} = .05).

Reward earnings trajectories for individual category ranks: Our primary analyses collapsed earnings across more (ranks 1 + 2) and less (ranks 3 + 4) preferred categories. This approach enhanced our statistical power. Compared to studies in mice and rats during Restaurant Row, we had relatively fewer datapoints per human subject (both in terms of the number of completed sessions, and the number of trials within each session). Figure S4 (*top*) shows video reward earning changes for each category rank individually (akin to the prior mouse studies, e.g., (Durand-de Cuttoli et al., 2025, 2024)). Here we constructed a linear-mixed effects model across all patients: Number of Videos Earned ~ Week X Category Rank + (Week|Patient). Results indicated a significant interaction (χ^2=12.22, p=.006), driven by modest increases in more preferred categories (t-ratio_{348_Rank1} = 2.13, p = .03, p_{adj} = .07; t-ratio_{348_Rank2} = 2.19, p = .03, p_{adj} = .07). Slopes for less preferred categories (Ranks 3 and 4) were not significant. The positive slopes for more preferred categories were also greater than those for less preferred categories (all ps and ps_{adj} < .05). Figure S4 (*bottom*) illustrates how changes in reward trajectories are driven by patients who showed reduced anhedonia following rTMS therapy. The corresponding linear-mixed effects model included a moderator variable to capture patients who showed improvements in anhedonia (binary variable): Number of Videos Earned ~ Week X Category Rank X Anhedonia Reduction + (Week|Patient). The three-way interaction was significant (χ^2=9.04, p=.03) and primarily driven by a slopes difference in reward earnings from Rank 3 when comparing patients who did and did not improve (t-ratio_{775_Rank3} = -2.52, p = .01, p_{adj} = .05).

Reward earnings trajectories beyond the 7-week mark: Our primary analyses were restricted to 7 weeks of treatment. However, several patients completed rTMS sessions beyond 7 weeks. Figure S5 shows that the overall trend for (more – less preferred) earnings, across all patients, remains when including data beyond 7 weeks (χ^2 = 4.45, p = .03). Follow-up tests revealed a significant, positive slope for more preferred earnings (χ^2 = 8.69, p = .003, p_{adj} = .006). The slope for less preferred earnings was not significant (p = .17).

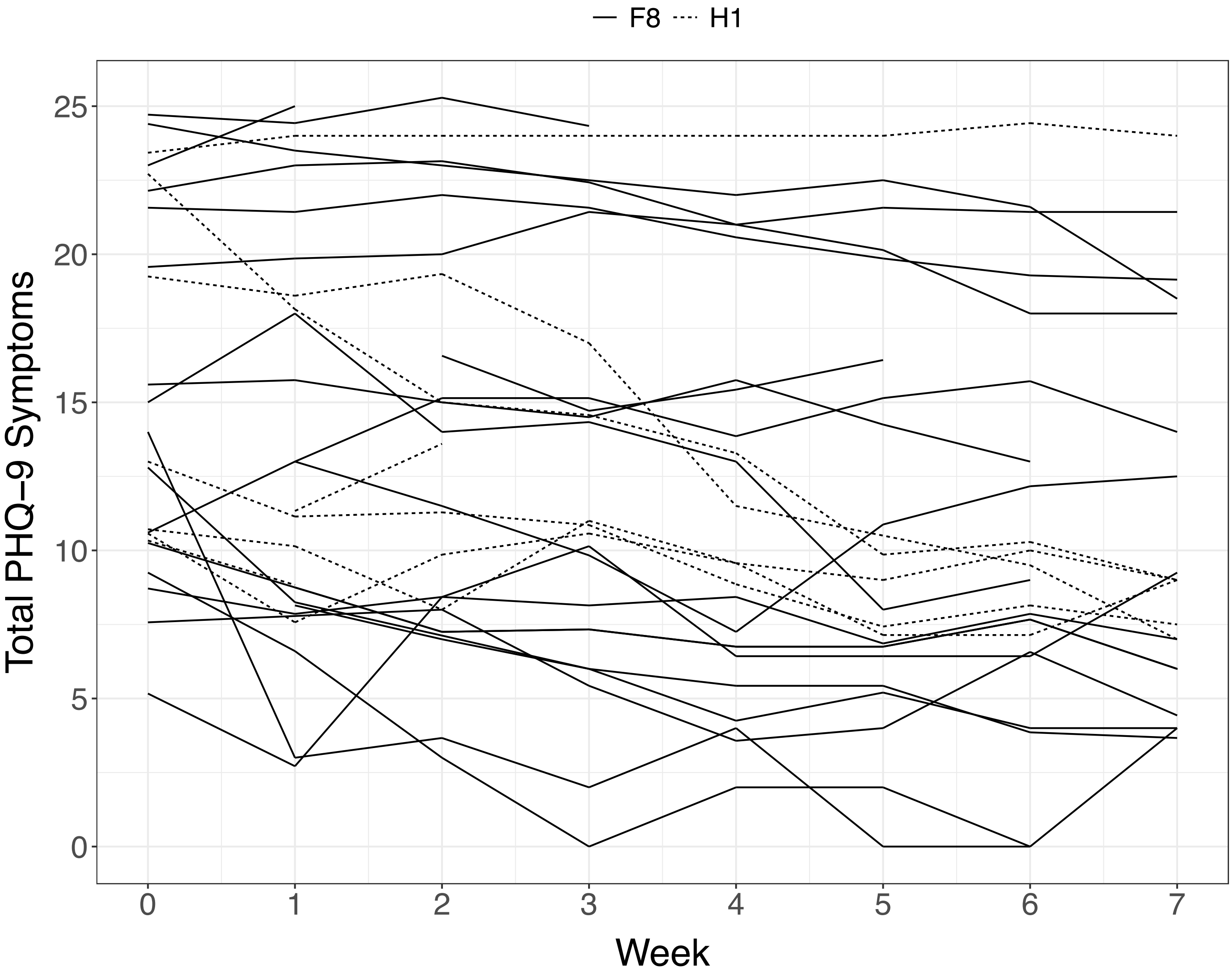

Figure S1. Depression symptoms declined over the course of repetitive transcranial magnetic stimulation treatment in patients with major depressive disorder. Each line represents an individual patient. The specific magnetic coil employed for that patient is represented by the solid (F8 coil) or dashed (H1 coil) lines.

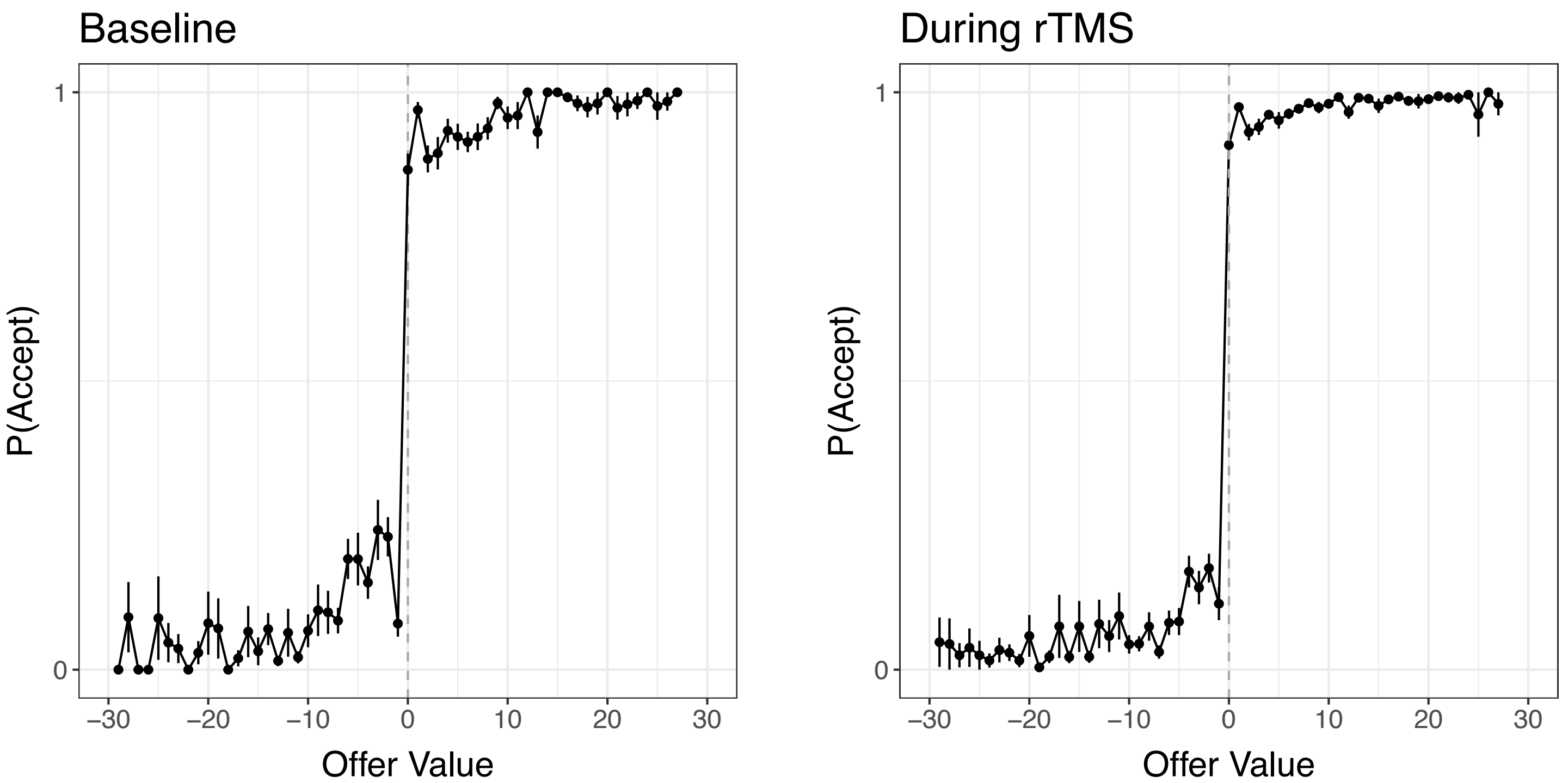

Figure S2. Decisions conformed to offer value in patients with major depressive disorder. Proportion of offers accepted as a function of offer value at baseline (*left*) and throughout rTMS treatment (*right*). Here a 1 = accept decision and 0 = reject decision, based on that offer's subjective value (i.e., category-specific delay threshold – offer delay. Offer values > 0 are considered high-value, likely to accept offers. Offer values < 0 are low value, and therefore less likely to accept offers. Data shown as the mean ± standard error of the mean across patients. Abbreviations: *rTMS* = repetitive transcranial magnetic stimulation.

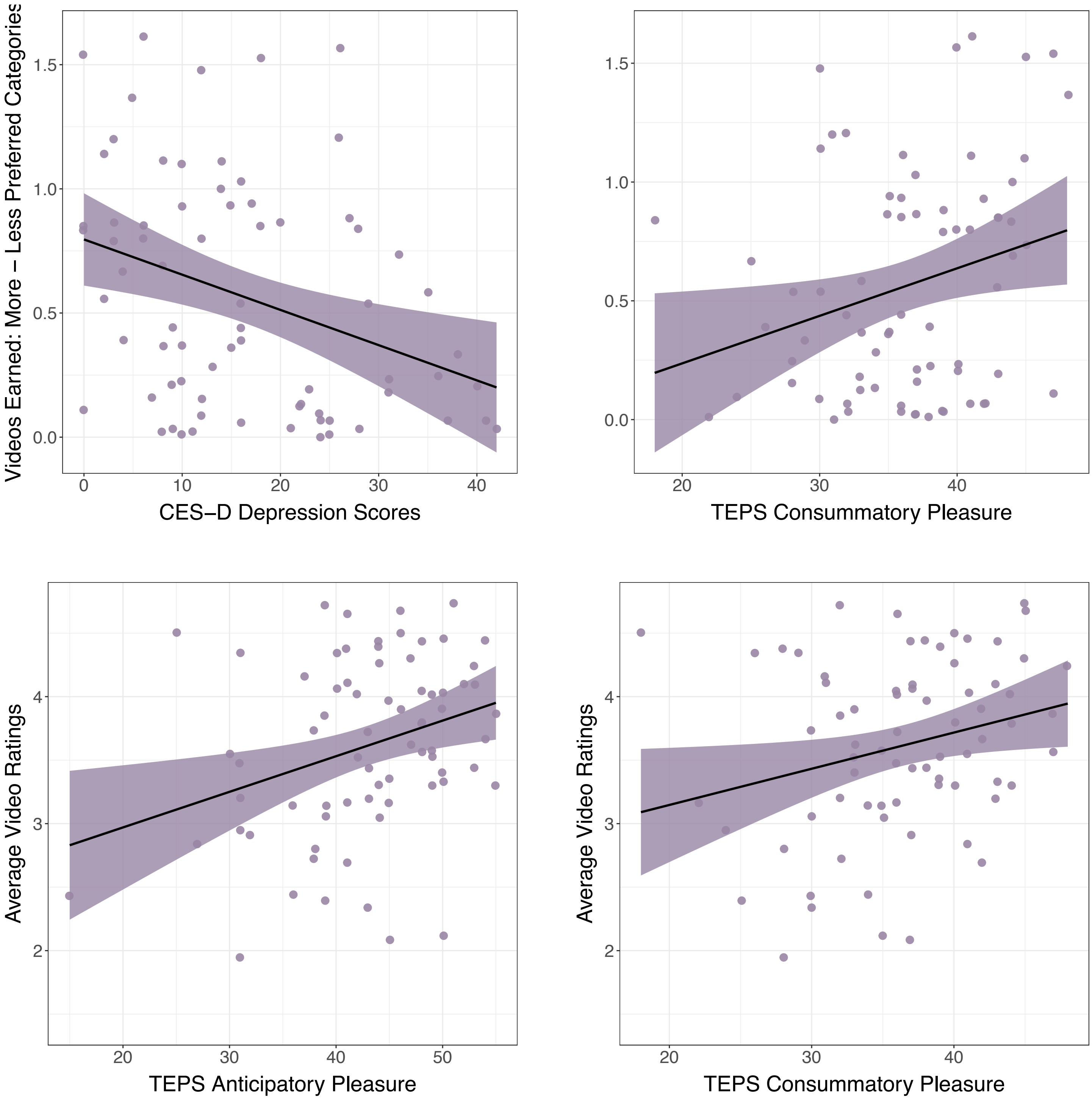

Figure S3. Correlations between reward earnings and hedonic ratings (on Web-Surf) with depression and pleasure ability from questionnaires. Reward earnings (more – less preferred) were negatively correlated with depression symptoms (*top left*) and positively correlated with consummatory pleasure ability (*top right*); though the latter effect did not survive multiple comparison correction. The positive correlation between more preferred earnings and consummatory pleasure ability was significant after correction (*not pictured*). Hedonic enjoyment measured based on video ratings positively correlated with anticipatory and consummatory pleasure ability (*bottom row*). Abbreviations: *CES-D* = Center for Epidemiological Studies – Depression; *TEPS* = Temporal Experience of Pleasure Scale.

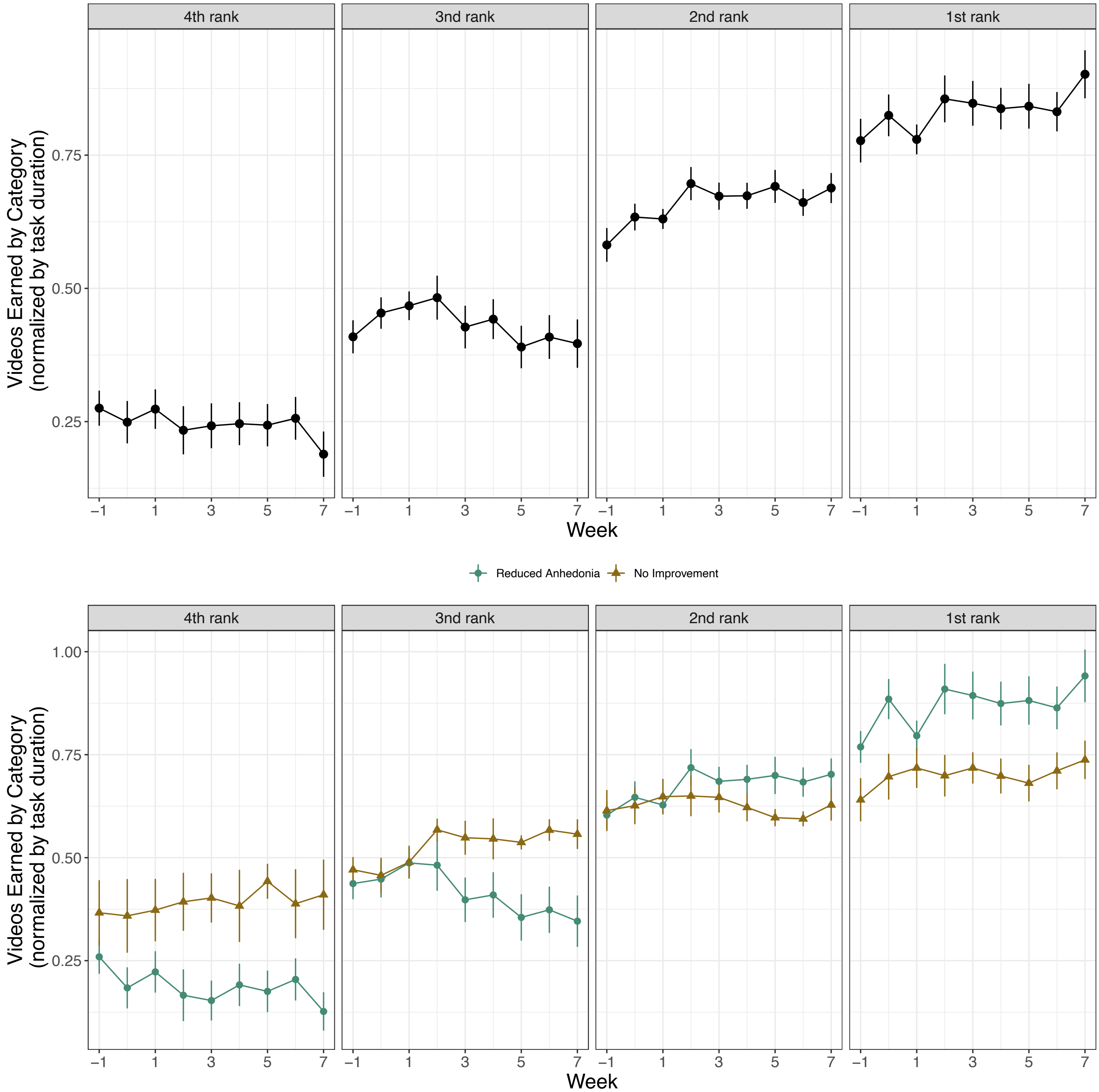

Figure S4. Patients with major depressive disorder increase their relative attainment of more preferred video rewards (Rank 1 and 2) over the course of transcranial magnetic stimulation (rTMS) therapy (*top*). Reward earning changes are driven by patients who show a reduction in anhedonia with rTMS; while those who did not improve show no change in earnings (*bottom*). Data shown as the mean ± standard error of the mean across patients. Vertical dashed line represents the start of rTMS. Abbreviations: *Tx* = treatment.

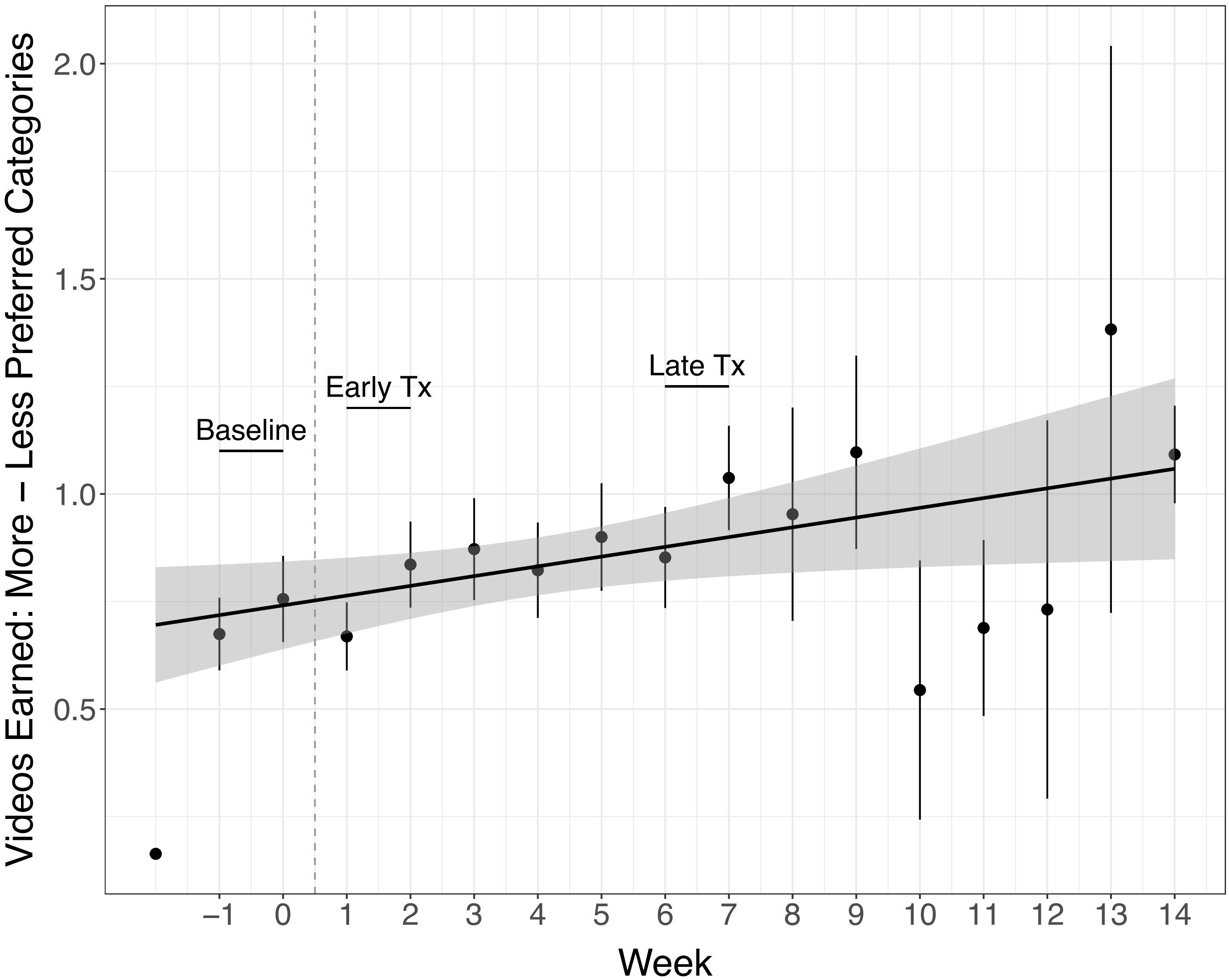

Figure S5. Patients with major depressive disorder increase their relative attainment of more preferred versus less preferred video rewards over the course of transcranial magnetic stimulation (rTMS) therapy. Data shown as the mean ± standard error of the mean across patients. Vertical dashed line represents the start of rTMS. Abbreviations: *Tx* = treatment.

Table S1. Clinical comorbidities (ICD10 codes) for patients with major depressive disorder

**ICD code range**	**Patients with a clinical comorbidity per code range** ***N*** **(%)**
Substance use disorders (F10 – F19)	3 (10%)
Primary psychoses (F20 – F29)	0 (0%)
Mood disorders (F30 – F39)	30 (100%)
Anxiety, stress, and somatoform disorders (F40 – F49)	19 (63%)
Behavioral disorders with physical factors (F50 – F59)	7 (23%)
Personality and behavioral disorders (F60 – F69)	2 (7%)
Intellectual disabilities (F70 – F79)	0 (0%)
Developmental disorders (F80 – F89)	2 (7%)
Behavioral and emotional disorders with onset in childhood (F90 – F98)	7 (23%)
Mental disorder, not otherwise specified (F99)	0 (0%)

Note: Diagnostic information missing for two patients.

Table S2. Medication data for patients with major depressive disorder

NbN System	*N* (%)	NbN MoA	*N* (%)
Serotonin	27 (87.10)	Reuptake inhibitor	9 (29.03)
		Receptor antagonist + reuptake inhibitor (SARI)	8 (25.81)
		Partial agonist	7 (22.58)
		Multimodal modulator	3 (9.68)
Catecholamine (DA/NE)	21 (67.74)	Reuptake inhibitor	14 (45.16)
		Release agent	7 (22.58)
Serotonin/Norepinephrine	19 (61.29)	Reuptake inhibitor	17 (54.84)
		Receptor antagonist	2 (6.45)
GABA	14 (45.16)	Positive allosteric modulator (PAM)	14 (45.16)
Ion channels	8 (25.81)	Sodium channel modulator	7 (22.58)
		Sodium channel blocker	1 (3.23)
Dopamine/Serotonin	6 (19.35)	Receptor partial agonist	4 (12.90)
		Receptor antagonist	2 (6.45)
Calcium channels	5 (16.13)	Alpha-2-delta ligand (gabapentoid)	5 (16.13)
Histamine	5 (16.13)	Receptor antagonist	5 (16.13)
Dopamine	4 (12.90)	Receptor agonist	3 (9.68)
		DAT inhibitor	1 (3.23)
Glutamate	1 (3.23)	Receptor antagonist	1 (3.23)
Orexin	1 (3.23)	Receptor antagonist	1 (3.23)

Note: Medication information was unavailable for one participant. % patients are reported proportionate to the 31 subjects for which pharmacological data were available. Percentages reflect proportion of patients with ≥1 exposure in the given category at the time of treatment. Mode-of-action percentages are calculated relative to the full cohort, not conditional on the NbN System.

Table S3. Laterality of target for repetitive transcranial magnetic stimulation (Figure-8 coil) for patients with major depressive disorder

	n
Left	17
Right	2
Both	7

Note: Some patients received a change in laterality during treatment.

Table S4. Treatment protocol breakdown for repetitive transcranial magnetic stimulation for patients with major depressive disorder

	1 Hz (*n*)	5 Hz (*n*)	10 Hz (*n*)	18 Hz (*n*)	[a]50 Hz (*n*)
F8 coil	3	1	2		15
H1 coil				11	

[a]50 Hz stimulation was equivalent to theta burst stimulation.

Table S5. Number of experimental sessions collected per week for patients with major depressive disorder

Week	*n*
-2	1
-1	26
0	26
1	28
2	27
3	29
4	29
5	28
6	28
7	26
8	11
9	6
10	4
11	7
12	4
13	2
14	2

References

Beam, W., Borckardt, J. J., Reeves, S. T., & George, M. S. (2009). An efficient and accurate new method for locating the F3 position for prefrontal TMS applications. *Brain Stimulation*, *2*(1), 50–54.

Durand-de Cuttoli, R., Issler, O., Pedersen, S. M. B., Yakubov, B., Jahan, N., Abid, A., … Sweis, B. M. (2025). Change-of-mind neuroeconomic decision-making is modulated by LINC00473 in medial prefrontal cortex in a sex-dependent manner. *Science Advances*, *11*(28), eadr3228.

Durand-de Cuttoli, R., Martínez-Rivera, F. J., Li, L., Minier-Toribio, A., Dong, Z., Cai, D. J., … Sweis, B. M. (2024). A double hit of social and economic stress in mice precipitates changes in decision-making strategies. *Biological Psychiatry*, *96*(1), 67–78.

Gard, D. E., Gard, M. G., Kring, A. M., & John, O. P. (2006). Anticipatory and consummatory components of the experience of pleasure: A scale development study. *Journal of Research in Personality*, *40*(6), 1086–1102.

Koo, T. K., & Li, M. Y. (2016). A guideline of selecting and reporting intraclass correlation coefficients for reliability research. *Journal of Chiropractic Medicine*, *15*(2), 155–163.

Kroenke, K., Spitzer, R. L., & Williams, J. B. W. (2001). The PHQ-9: Validity of a brief depression severity measure. *Journal of General Internal Medicine*, *16*, 606–613.

Mcinnes, A. N., Sullivan, C. R. P., MacDonald III, A. W., & Widge, A. S. (2025). Psychometric validation and preliminary clinical correlation of an experiential foraging task. *Assessment*, 10731911251376214.

Nusslock, R., Mittal, V. A., & Alloy, L. B. (2025). reward processing in mood disorders and schizophrenia: A neurodevelopmental framework. *Annual Review of Clinical Psychology*, *21*(1), 557–584.

Patton, J. H., Stanford, M. S., & Barratt, E. S. (1995). Factor structure of the barratt impulsiveness scale. *Journal of Clinical Psychology*, *51*(6), 768–774.

Radloff, L. (1977). The CES-D scale: A self-report depression scale for research in the general population. *Applied Psychological Measurement*, *1*(3), 385–401.

Sackeim, H. A., Aaronson, S. T., Bunker, M. T., Conway, C. R., Demitrack, M. A., George, M. S., … Rush, A. J. (2019). The assessment of resistance to antidepressant treatment: Rationale for the Antidepressant Treatment History Form: Short Form (ATHF-SF). *Journal of Psychiatric Research*, *113*, 125–136.

Sonmez, A. I., Webler, R., Krueger, A. M., Godoy-Henderson, C., Sullivan, C., Wilson, S., … Albott, C. S. (2024). Effects of TMS on anhedonia and suicidal ideation in treatment-resistant depression: Outcomes from the University of Minnesota Interventional Psychiatry Program. *Journal of Mood & Anxiety Disorders*, *8*, 100073.
